# Estimated risk of HIV acquisition and practice for preventing occupational exposure: a study of healthcare workers at Tumbi and Dodoma Hospitals, Tanzania

**DOI:** 10.1186/1472-6963-13-369

**Published:** 2013-09-30

**Authors:** Kijakazi O Mashoto, Godfrey M Mubyazi, Emmanuel Makundi, Hussein Mohamed, Hamisi M Malebo

**Affiliations:** 1 National Institute for Medical Research, P.O. Box 9653, Dar es Salaam, Tanzania; 2 Muhimbili University of Health and Allied Sciences, P.O. Box 65000, Dar es Salaam, Tanzania

**Keywords:** HIV, Occupational exposure, Healthcare workers

## Abstract

**Background:**

Healthcare workers (HCWs) are at risk of acquiring human immuno-deficiency virus (HIV) and other infections via exposure to infectious patients’ blood and body fluids. The main objective of this study was to estimate the risk of HIV transmission and examine the practices for preventing occupational exposures among HCWs at Tumbi and Dodoma Hospitals in Tanzania.

**Methods:**

This study was carried out in two hospitals, namely, Tumbi in Coast Region and Dodoma in Dodoma Region. In each facility, hospital records of occupational exposure to HIV infection and its management were reviewed. In addition, practices to prevent occupational exposure to HIV infection among HCWs were observed.

**Results:**

The estimated risk of HIV transmission due to needle stick injuries was calculated to be 7 cases per 1,000,000 HCWs-years. Over half of the observed hospital departments did not have guidelines for prevention and management of occupational exposure to HIV infections and lacked well displayed health and safety instructions. Approximately, one-fifth of the hospital departments visited failed to adhere to the instructions pertaining to correlation between waste materials and the corresponding colour coded bag/container/safety box. Seventy four percent of the hospital departments observed did not display instructions for handling infectious materials. Inappropriate use of gloves, lack of health and safety instructions, and lack of use of eye protective glasses were more frequently observed at Dodoma Hospital than at Tumbi Hospital.

**Conclusions:**

The poor quality of the hospital records at the two hospitals hampered our effort to characterise the risk of HIV infection acquisition by HCWs. Greater data completeness in hospital records is needed to allow the determination of the actual risk of HIV transmission for HCWs. To further reduce the risk of HIV infection due to occupational exposure, hospitals should be equipped with sufficient personal protective equipment (PPE) and HCWs should be reminded of the importance of adhering to universal precautions.

## Background

The World Health Organization (WHO), International Labor Organization (ILO) and their allied organizations/institutions recognise the need for placing exposure to HIV as a workplace priority intervention. According to the WHO, the term ‘exposure to HIV’ is defined as ‘percutaneous injury (e.g. needle-stick; or cut with a sharp object) or the contact of mucous membrane or non-intact skin (e.g. exposed skin that is chapped, abraded or afflicted with dermatitis) with blood, tissue or other body fluids that are potentially infectious’ [1]. Empirical evidence has shown that HCWs are among the most at risk groups for contracting the HIV virus through blood and body fluids from their workplace which may lead to AIDS – Acquired Immune Deficiency Syndrome [2]. Apart from the chronic illness the HCW faces as a result of contracting HIV, the possibility of persistent recurrent nightmares and fear after one’s exposure to HIV cannot be underrated [3]. Such exposures can have a negative impact on families and colleagues of the HCW [4].

The risk of pathogen transmission from infected persons through an injury with a sharp object has been estimated to be 0.3% for HIV [5]. Developing countries, especially those in sub-Saharan Africa (SSA) account for the highest prevalence of HIV-infected patients and that 90% of occupational exposure occur in these countries [6-9]. Shortage of occupational safety facilities and HCWs’ sensitiveness to taking precautionary measures are among the reasons contributing to high frequency of occupational exposure in these countries [10].

Ensuring that safety devices are in place and workplace safety practices are adhered to in order to help prevent acquisition of infection is greatly recommended [11]. According to experts, safety devices and practices include: routinely using barriers such as gloves and goggles when anticipating contact with blood or body fluid; immediately washing hands and other skin surfaces after contact with blood or body fluid; carefully handling and disposing sharp instruments during and after use, and administering post-exposure prophylaxis [1]. Post-exposure prophylaxis alone is said to reduce sero-conversion by 81% [6].

The nature (including the source of infections) and the actual magnitude of the exposed HCWs, including the prevalence of exposure events and number of HCWs exposed is inadequately reported from most of sub-Saharan African countries and this is partly due to the lack of systematic studies [1]. In Tanzania, documented information on the estimated risk of occupational exposure to HIV infection among HCWs is scarce and this means more systematic evidence is needed. Therefore, the main objective of this study was to estimate the risk of HIV acquisition after occupational exposure and examine HCWs practices for preventing occupational exposure to HIV infection at Tumbi and Dodoma Hospitals, Tanzania.

Dodoma is a government owned hospital with the bed capacity of 420 and serves 118,000 Patients per year as a regional referral hospital. Tumbi is a parastatal owned hospital with 253 beds and serves as a designated regional referral hospital to 300,000 patients per year. Both hospitals are located in urban areas and provide inpatient and outpatient services such as general OPD, care and treatment clinic (CTC), voluntary counselling and testing (VCT), reproductive and child health (RCH) and clinics for Tuberculosis and Leprosy, Diabetes, eye, dental, gynaecology and obstetrics, etc.

## Methods

### Study population

The study population was defined as health workers at risk of percutaneous and muco-cutaneous injuries. This included physicians, dentists, nurses, midwives, laboratory technicians, nursing aids and medical assistants. Others included support staff such as laundry workers and cleaners.

### Study design

The study was carried out at two tertiary hospitals, namely Tumbi Designated Regional Hospital in Coast region and Dodoma Regional Hospital in Dodoma region. Tumbi Hospital was selected because of its popularity of frequently attending patients involved in car road accidents along highway roads to Morogoro region, Segera, Moshi and Arusha, and Dar es Salaam [12]. The road accidents factor was considered in the design of this study due to the fact that in such a situation, HCWs are often caught unprepared and there may be an increased risk of exposure to HIV among HCWs when attending injured patients. The Dodoma Hospital, being in the central region in Tanzania, was purposefully selected to represent other regions in the country which experienced regular or few incidents of road accidents.

This study was conducted in two parts. The first part of the study involved case record review of reported occupational exposures. The second part of the study was to observe HCWs’ practices for preventing occupational exposures. Data was collected in the months of February and March in 2012.

### Data collection techniques

#### Review of reported occupational exposures

Data on the incidence of occupational exposures were obtained through a desk review of the hospital records documented between January 2005 and May 2011. The initial plan was to review the records for the period 1998–2011, but a pilot survey for this study confirmed that there was no data for the years 1998 and 2004 therefore January 2005 was selected as the new start date for the review. Specifically, the information collected was related to the number of: HCWs, needle-stick injuries, sustained wound cuts with a sharp object, non-intact skin exposures, mucous membrane exposures (splashes), and bite injuries resulting in blood exposure to either person involved. Additional data were related to: total number of occupational exposures to HIV infection and number of HCWs who received post exposure prophylaxis (PEP). In order to determine HIV incidence after occupational exposure, data on HIV test results for HCW at the time of exposure and at 3 months post exposure was also collected. In both hospitals the exposed HCWs normally report the incident to a specific focal person who provides voluntary counseling and testing (VCT) services, and PEP when indicated. The exposed HCW whose HIV test is negative is normally followed up for a six months period.

#### Observation of HCW practices for preventing occupational exposures

Using an observational check list, an observation was made to the HCWs on whether in their service delivery practices they were adhering to the recommended measures for preventing themselves or their patients from occupational exposure to HIV infection. The research team conducting the observations took notes on the following aspects: if there was well displayed relevant HIV/AIDS information in service delivery places (including posters, signs or instructions for health and safety measures), availability of standard operating procedures including patient, equipment and supplies handling protocols during and after service delivery, policy guidelines for prevention of occupational exposure to HIV, availability of PPE such as gloves, boots, aprons and eye protective glasses. Attention was paid to whether and how the HCWs protected themselves while performing their duties and how hazardous/infectious waste materials were being handled and disposed as per recommendations [1]. A total of 12 departments at Tumbi Hospital and 11 departments at Dodoma Hospital, respectively, were visited to observe HCWs’ practice in relation to prevention of occupational exposure to HIV at the workplace. The specific sections visited at each Hospital were the: dental clinic, incineration unit, laboratory, labour ward, laundry, medical ward, mortuary, outpatient department, paediatric ward, surgical ward and operating theatre. At Dodoma Hospital, the Reproductive and Child Health (RCH) Clinic was also visited for observation purposes.

### Ethical considerations

Ethical clearance was obtained from the Medical Research Coordinating Committee (MRCC) through National Institute for Medical Research (NIMR) Secretariat. Permission to undertake the study at the selected hospitals was obtained from Regional Medical Officers (RMOs) and Council Medical Officers of Health (CMOH) of the respective regions and Municipal Councils of Dodoma and Kibaha. The study respondents/participants (HCWs) were also asked to choose to participate voluntarily after getting clear message using a special informed consent process sheet.

### Data analysis

There were no data available to confirm any HIV infection due to occupational exposure we therefore estimated the risk using information such as prevalence among the hospital patient population, probability of sero-conversion following exposure and PEP efficacy from other publications. The exposed population was defined as healthcare and other workers who had at least one percutaneous or muco-cutaneous injury in the period between 2005 and 2011. The risk of HIV transmission following percutaneous injury was estimated using the following data: number of HCWs at risk, the number of occupational exposures, and the prevalence of active infection in the patient population and the risk of sero-conversion per incident. Thus to estimate the risk of HIV transmission due to occupational exposure, we multiplied the HIV prevalence among the hospital population, the number of exposure incidents over a period of time, and the risk of sero-conversion per incident [13,14]. The PEP efficacy of reducing HIV transmission by 81% following occupational exposure was also applied to the formula [6]. As there were no data for number of HCWs at risk for Dodoma in year 2006–2008, we used data from year 2009 – 2011 to get the average number of HCWs at risk per year. We then multiplied the average number of HCWs at risk by six (years of observation) to get HCW years for Dodoma hospital. Hospitals’ differences in the practice for preventing occupational exposure between the two hospitals were tested using Chi- Square statistical test.

## Results

### Incidence of occupational exposure, PEP compliance and estimated risk of HIV transmission for HCWs

The study data were available from Dodoma Hospital for the period of 2006–2011. The number of needle stick injuries ranged from 1–11 per annum. Data relating to the number of HCWs at risk of occupational exposure were available for the years 2009, 2010 and 2011only (Table 1).

**Table 1:** Cases of occupational exposure, number of HCWs at risk, HIV status and use of PEP at Dodoma Hospital

	**2006**	**2007**	**2008**	**2009**	**2010**	**2011**
**Type of exposure**						
Needle stick injuries	1	2	4	4	11	8
Sharp object/equipment injuries	ND	ND	2	ND	2	ND
Blood or body fluid splashes	ND	ND	ND	1	ND	1
Number of HCW at risk	ND	ND	ND	345	372	391
**HIV status and use of PEP**						
Exposed HCW tested for HIV	1	2	6	5	13	9
Exposed HCWs who received PEP	1	2	6	5	13	9
Exposed HCWs who tested after PEP	ND	ND	ND	ND	ND	ND
Exposed HCWs who seroconvert	ND	ND	ND	ND	ND	ND

*ND* No data.

Data for the period 2006–2011 was retrieved from Tumbi Hospital. The hospital recorded 22 cases of needle stick injuries. The Hospital had no data on splashes and injury by sharp object/instrument (Table 2).

**Table 2:** Cases of occupational exposure, number of HCWs at risk, HIV status and use of PEP at Tumbi Hospital

	**2005**	**2006**	**2007**	**2008**	**2009**	**2010**	**2011**
**Type of exposure**							
Needle stick injuries (NSIs)	4	7	5	4	2	0	0
Sharp object/equipment injuries	ND	ND	ND	ND	ND	ND	ND
Blood or body fluid splashes	ND	ND	ND	ND	ND	ND	ND
Number of HCWs at risk	352	352	354	353	355	358	360
**HIV status and use of PEP**							
Exposed HCWs who tested for HIV	4	7	5	4	2	0	0
Exposed HCWs who received PEP	4	7	0	0	0	0	0
Exposed HCWs who tested after PEP	ND	ND	ND	ND	ND	ND	ND
Exposed HCWs who seroconvert	ND	ND	ND	ND	ND	ND	ND

*ND* No data.

Sources are not routinely tested but assumed to be HIV positive for the purpose of PEP prescription. At both hospitals, initial HIV test results were negative for all exposed HCWs. Although PEP was initiated to almost all exposed HCWs, no HCW returned for follow up testing and there were no data on PEP compliance and HIV test results subsequent to PEP initiation.

The occupational risk calculation will consider only needle stick injury due to low numbers of incidences caused by other means of occupational exposure such as splashes, sharp object/instrument etc. From 2005 to 2011, on average a total of 58 cases of occupational exposure were recorded in the two hospitals, (89.6%) mainly due to needle stick injuries (Table 3).

**Table 3:** Frequency and percent distribution of occupational exposures in Tumbi and Dodoma Hospitals

**Occupational exposure**	**2005**	**2006**	**2007**	**2008**	**2009**	**2010**	**2011**	**Total**	**%**
All types	4	8	7	10	7	13	9	58	100
Needle stick injuries	4	8	7	8	6	11	8	52	89.6
Sharp object/equipment injuries	ND	ND	ND	2	ND	2	ND	4	7.0
Blood or body fluid splashes	ND	ND	ND	ND	1	ND	1	2	3.4

*ND* No data.

The annual incidence of needle stick injuries was 0.011 per HCW years, and the estimated risk of HIV acquisition due to needle stick injuries was calculated to be 7 cases per 1,000,000 HCW-years (Table 4).

**Table 4:** Estimated risk of HIV infection due to needle stick injuries (Cases per 1,000,000 HCW-years)

**Hospital**	**Cases**	**HCW-years**	**Incidence per HCW-years**	**Estimated risk**
Dodoma	30	2,216	0.0135	8
Tumbi	22	2,484	0.0088	5
All	52	4,701	0.0110	7

### Practice of preventing occupational exposure to HIV infection

Seventy four percent of the Hospital departments observed did not have displayed instructions for handling infectious materials. For instance, more than half of the observed sections lacked the guidelines for prevention and management of occupational risk of exposure to HIV infection and well displayed health and safety instructions. Of the 72% of the Hospital departments observed, the HCWs found did not appropriately use colour coded plastic containers for disposal of waste materials. Moreover, the personal protective equipment was not consistently worn by the HCWs. Inappropriate use of gloves was more frequently observed at Dodoma Hospital than at Tumbi Hospital. Fourteen out of twenty three Hospital departments observed, the HCWs did not wear protective goggles (Table 5).

**Table 5:** Number and percentage of Hospital sections not adhering to the guidelines for preventing occupational exposure to HIV infection (N = 23)

**Practice**	**Tumbi Hospital sections**	**Dodoma Hospital sections**	**Total Hospital sections**
	**N (%)**	**N (%)**	**N (%)**
Lack of health and safety signs/posters/instructions	5 (41.7)	8 (72.7)	13 (56.5)
Lack of protocol/guidelines for prevention and management of occupational exposure to HIV	7 (58.3)	5 (45.5)	12 (52.2)
Inappropriate use of colour coded plastic bags/containers/safety boxes for waste materials	1 (8.3)	4 (36.4)	5 (21.7)
Poor hand washing (not washing at all or washing after procedure only)	2 (16.7)	1 (9.1)	3 (13.0)
Inappropriate use of gloves	1 (8.3)	8 (72.7)*	9 (39.1)
Not wearing boots	4 (33.3)	3 (27.3)	7 (30.4)
Not wearing aprons	6 (50.0)	5 (45.5)	11 (47.8)
Not wearing protective eye glasses	7 (58.3)	7 (63.6)	14 (60.9)
Lack of displayed instructions for handling infectious/hazardous materials	10 (83.3)	7 (63.6)	17 (73.9)

*P = *0*.*003* (*Chi*-*Square test*).

## Discussion

A total of 58 cases of occupational exposure were recorded at 2 hospitals over a 6 year period. Of these, 52 were NSIs and the incidence of NSIs was 0.011 per HCW year. The follow up data on HIV status of exposed HCWs were not available as none of the HCW returned for a follow up test. Adequate handling of blood exposure accidents requires reporting of the accidents by the victims [15]. Studies in other hospitals worldwide indicate that only half of occupational exposure accidents are actually reported [8]. In this study the number of unreported incidents, HIV status after PEP initiation and compliance to prescribed PEP is not known. Thus the quality of data collected is questionable and there is the possibility that the risk of acquiring HIV infection from needle-stick injuries reported in this study is underestimated. Furthermore, this study did not estimate the prevalence of HIV in hospital patient population, and thus used hospital HIV prevalence reported in other hospitals in Tanzania [16]. This possibly has led to over or underestimation of the risk of acquiring HIV infection due to occupational exposure.

When a HCW reports an exposure, sources are not routinely tested but assumed to be HIV positive for the purpose of PEP prescription. Exposed HCWs are scheduled for follow up appointments at 3 and 6 months subsequent to incident of occupational exposure to HIV. However, there is no mechanism in place to check whether HCWs took PEP as prescribed. Gaps in the documentation of the health service management data at the studied health facilities are reflected in Tables 1 and 2. The number of unreported accidental exposures is not known. Follow up data on the HIV status of the exposed HCWs after PEP or 3 months after the occupational exposure were not available mainly due to the fact that the exposed HCWs did not return for the second HIV test as per requirement. Therefore, we were not able to discern any incidence of acquisition of HIV infection due to occupational exposure. In all probabilities another factor relating to non-attendance of the required second HIV test might be “self-counselling” which frequently takes place, and this means that HCWs decide not to go for the second test because the overall risk involved is considered to be low.

Using the risks of sero-conversion after percutaneous exposure of 0.32% [17], a presumed HIV prevalence of 24% among hospital patients in Tanzania [16] and an incidence of 0.011 needle-stick injuries per HCW-years and taking into the account that all exposed HCWs were offered PEP, the estimated risk of HIV infection due to needle stick injury is 7 cases per 1,000,000 HCW-years. The calculation based on the assumption that all exposed HCWs complied with the prescribed PEP. However, it is not known how many, if any, actually took the PEP or completed the course. This means, the calculated risk may be underestimated and therefore should be interpreted with caution. If none of the exposed HCW took the prescribed PEP, the estimated risk would have been 8 cases per 1,000,000 HCW-years. If the number of needle-stick injuries doubled the estimate would have been 16 cases per 1,000,000 HCW-years. If the number of needle-stick injuries were four times the number recorded, the estimated risk of HIV transmission would have increased to 34 cases per 1,000,000 HCW-years. Nevertheless potential variability of degree of exposure (i.e. seriousness or severity of exposure, quantity of transmitted blood or contaminated material) was not taken into account. This might vary from one discipline to another, e.g. between internal medicine and surgery. Furthermore, because of lack of data, it was not clear whether multiple needle stick occurrences involved the same patient.

The most striking thing about the results from the present study is the very low number of incidents reported. Given the WHO calculation of 1/300,000 worldwide [9], then one would expect a much higher risk for HCWs in sub Saharan Africa (SSA). Therefore the calculated risk of 7/1,000,000 HCW years is likely to be a gross underestimate. Poorly implemented surveillance of occupational exposure to HIV in the two Hospitals may explain this gross underestimation. Nevertheless, under reporting of blood and body fluid exposure is common because of a belief that most exposures are not significant [18]. In contrary 91% of junior doctors in South Africa reported a needle-stick injury in the previous 12 months [19]. However, occurrence of occupational exposure among interns is very common [18] and these junior doctors were frequently motivated to report needle-stick injuries they encountered [19].

The estimated risk of HCWs acquiring HIV infection following needle stick injury reported in the present study is lower than the incidence of HIV infection calculated in a study conducted in 1997 in Mwanza region [14]. With an average of 5 percutaneous injuries per HCW per year, 20% HIV prevalence among patients and a transmission probability of 0.25%, the Mwanza study calculated the incidence of HIV infection through occupational exposure to be 0.27% or approximately 3 per 1000 HCWs per year [14]. The present study estimated the risk of HIV infection using needle-stick injuries only, excluding other percutaneous injuries caused by sharp object or instrument. In addition implementation of universal precautions to infection at these health care facilities may explain the reduced risk of HIV transmission from the patient to the HCW and vice versa. The Mwanza study was conducted prior to implementation of universal precautions.

Prevention of HIV in healthcare settings requires preventive measures towards exposure to blood and blood products and the availability of PEP [20]. However, the use of PEP should not be the primary means to prevent occupationally acquired HIV infection. In countries with a high incidence of percutaneous injuries and high prevalence of HIV, PEP use may become difficult because of the related high cost and toxicity of the drugs. An important tool to protect HCWs against occupational HIV infection is to follow standard universal precautions (e.g. use of PPE, and the use of safety devices such as retractable needles. These devices are a suitable and important tool in the reduction of needle stick injuries and improving medical staff’s health and safety [13,21].

Hygiene measures taken by Dodoma and Tumbi Hospitals to reduce the risk of infection were insufficient. Over 50% of the Hospital departments at the studied Hospitals did not have health and safety guidelines/instructions in place at all or in clearly visible places for quick reference. Lack of health and safety instructions were more frequently observed at the Dodoma Hospital than at Tumbi Hospital. At both Hospitals, measures for preventing occupational exposures to HIV infection are poorly implemented. A good example is the inappropriate use of gloves. This poor practice seems to have partly been contributed by lack of essential working facilities. Use of PPE such as boots, aprons and eye protective glasses depended on their availability and sometimes HCWs shared torn aprons. In some Hospital departments, the hand washing practice was hampered by lack of running tap water and towels. Lack of towels forced HCWs to dry their hands on the clothes they are wearing or hanging curtains.

Crucial to prevention of occupational exposure to infectious diseases is the handling of infectious materials. Instructions for handling infectious materials were not displayed in most of the hospital departments observed. Though plastic bags/containers/safety boxes for waste disposal were colour coded, approximately one-fifth of the hospital departments visited failed to adhere to the instructions pertaining to correlation between waste materials and the corresponding colour coded bag/container/safety box.

The present study has been able to identify specific needs and concerns for action in the current surveillance system of occupational exposure to HIV. More information is needed from all levels of healthcare especially, lower level facilities such as primary and secondary healthcare facilities. These facilities may be even more poorly supported with the necessary human resources, equipment etc. compared to tertiary hospitals. Information from the private sector is needed to add to the database as a necessity for advocacy and improvement.

## Conclusions

Although the estimated risk of HIV infection at the studied hospital settings is low, the practice of prevention measures to infection by the HCWs is inadequate and needs to be strengthened. The government’s prospects for seeing all health service delivery sections have all the necessary facilities to support the service providers in relation to HIV prevention seems not to be supported by a true picture on the ground. The quality of hospital records on occupational exposure to HIV infection at the two hospitals was found to be lacking and thus indicates the need to improve the current reporting systems. Better data completeness in hospital records is needed to allow determining the actual risk of HIV transmission for HCWs. To further reduce the risk of HIV infection due to occupational exposure, hospitals should be equipped with sufficient personal protective equipment and HCWs should be reminded and re-trained periodically on universal precautions and the importance of adhering to them. Further study should explore reasons why exposed HCWs do not return for second HIV test after occupational exposure.

## Abbreviations

AIDS: Acquired Immunodeficiency Syndrome; HCWs: Health Care Workers; HIV: Human Immunodeficiency Virus; MRCC: Medical Research Coordinating Committee; NIMR: National Institute for Medical Research; PEP: Post Exposure Prophylaxis; PPE: Personal Protective Equipment; TANHER: Tanzania Health Research Forum; WHO: World Health Organization.

## Competing interests

The authors declare that they have no competing interests.

## Authors’ contributions

KOM: Principal investigator, conceived of the study, designed the study, collected data, statistical analysis and manuscript writing. GMM: designed study and manuscript writing. HMM: Participated in design of study and manuscript writing. HM: collected data and manuscript writing. EM: Have commented on the paper and provided valuable guidance for manuscript write up. All authors read and approved the final manuscript.

## Pre-publication history

The pre-publication history for this paper can be accessed here:

http://www.biomedcentral.com/1472-6963/13/369/prepub
